# Trends and factors associated with anemia among women of reproductive age in Mali: analysis of data from 2001 to 2018 Mali demographic and health surveys

**DOI:** 10.1186/s13690-025-01637-w

**Published:** 2025-07-31

**Authors:** Ebenezer Kwesi Armah-Ansah, Eugene Budu, Charity Oga-Omenka, Marina Kolosnitsyna

**Affiliations:** 1 School of Public Health Sciences, Faculty of Health, University of Waterloo, Waterloo, Canada; 2 Research Unit, Korle Bu Teaching Hospital, P. O. Box, 77, Accra, Ghana; 3 Department of Applied Economics, National Research University - Higher School of Economics, Moscow, Russia

**Keywords:** Anemia, Reproductive age, Mali, Prevalence, Trends, Women’s health

## Abstract

**Background:**

Even though widespread attempts have been made to halve the incidence of anemia by 2025, the prevalence remains alarmingly high among women of reproductive age in Africa. The government of Mali has underscored the necessity for a multi-sectoral approach to reducing the anemia burden. However, no studies have considered the trends and factors associated with anemia among women of reproductive age in Mali. Hence, this study examined the prevalence and trends of anemia among women of reproductive age in Mali from 2001 to 2018.

**Methods:**

The study analyzed datasets from the 2001, 2006, 2012–2013, and 2018 Mali Demographic and Health Surveys. We included a total weighted sample of 18,096 women of reproductive age who had participated in the hemoglobin test. Multilevel regression analysis was employed, and the results were presented as adjusted odds ratio (aOR), with 95% confidence intervals (CIs) and *p*-values used to show the results.

**Results:**

We found that anemia prevalence in Mali remained persistently high, with a marginal increase from 63.4% in 2001 to 63.5% in 2018. The overall prevalence of anemia among women of reproductive age in Mali from 2001 to 2018 was 59.3% [95% CI: 58.2–60.4%]. We found, however, that about 2.6% were severely anemic. The study found that women of reproductive age who have secondary and above education [aOR = 0.75, 95% CI = 0.67–0.84], overweight women [aOR = 0.62, 95% CI = 0.55–0.70], women in the richest wealth quintile [aOR = 0.83, 95% CI = 0.71–0.97], women who have improved sources of drinking water [0.91, 95% CI = 0.84–1.10], women who lived in the Kidal Region [aOR = 0.38, 95% CI = 0.30–0.49], and women of reproductive age who lived in communities with medium literacy [aOR = 0.80, 95% CI = 0.73–0.87] had lower odds of being anemic. However, women who were captured in the 2018 survey year [aOR = 1.23, 95% CI = 1.11–1.37], women with four or more births [aOR = 1.15, 95% CI = 1.02–1.30], and women who were pregnant [aOR = 1.57, 95% CI = 1.42–1.73] had higher odds of developing anemia.

**Conclusion:**

The prevalence of anemia among women of reproductive age in Mali remains alarmingly high and virtually unchanged in 17 years. To address this persistent public health challenge, coordinated action is necessary. The government and public administrators should prioritize strengthening antenatal care services through free maternal health care, iron supplementation, treated mosquito nets, and deworming programs. Additionally, the Ministry of Water and Sanitation, in collaboration with traditional leaders and community health authorities, must ensure that every household has access to protected, potable drinking water. Finally, the Ministry of Education and the Ministry of Health and Public Hygiene should jointly develop targeted health and nutrition interventions and programs that would mitigate anemia prevalence among women of reproductive age in Mali. Without these concerted efforts, this significant public health burden is likely to persist.

**Table:** 

Text box 1. Contributions to the literature	
• This study stresses the importance of comprehensive interventions, such as free maternal health care, the provision of iron supplements, treated mosquito nets, and deworming, and educational initiatives, to tackle the enduringly high rates of anemia in Mali, in line with the Sustainable Development Goals (SDGs). • The research identifies considerable regional variations in anemia prevalence, underscoring the necessity for focused, context-relevant interventions to address the diverse risk factors present in different areas of Mali. • This study adds to the global understanding of anemia by presenting data from a low-income, high-burden country, providing insights that could guide similar initiatives in other African and Asian countries.	

## Introduction

Anemia is characterized by a low level of healthy red blood cells, which prevents the body from carrying adequate oxygen to the heart, brain, and muscles [1]. The reduction in the healthy red blood cells is hence termed anemia [2]. Anemia among women who are in their reproductive age is diagnosed when the “hemoglobin” (Hb) concentration falls below 11.0 grams per deciliter (g/dl) among those who are pregnant and below 12.0 g/dl among non-pregnant women,” as per the World Health Organization (WHO) guidelines [1, 3–5].

Anemia affects all ages but is more prevalent in children and women of reproductive age (WRA) [4]. Anemia is caused by a mixture of characteristics connected to women’s physiological make-up and specific socioeconomic settings. A combination of environmental, socioeconomic, and physiological variables affects these characteristics. These consist of the drinking water quality, the state of sanitation, the number of children, the literacy rate in the community, and the socioeconomic level [6, 7]. Anemic women may also suffer from several other health complications, such as genetic blood disorders, chronic diseases, iron deficiency, and inadequate nutrient intake [7, 8].

A substantial number of people around the globe, amounting to over a billion, are believed to have been affected by anemia [9, 10]. When the prevalence of this medical condition exceeds 5% of the population, it significantly impacts public health [10]. Over the past 20 years, there has been minimal progress in decreasing the anemia prevalence among WRA, with some countries in the South Asia and Africa regions even witnessing an increase [11–13]. Interestingly, these regions collectively account for approximately 89% of the global anemia burden [11]. Within developing countries, the occurrence of anemia varies by location, with rural areas displaying a higher percentage (44.3%) than urban regions (40.2%) [6]. More recent data from WHO suggests that the prevalence of anemia in WRA globally rose from 30 to 33% between 2011 and 2016 [13]. In developed nations like Australia and the United States, anemia rates among WRA are reported at 20% and 18%, respectively; in contrast, developing countries show much higher figures, such as 50.1% in Ethiopia, 76.7% in Pakistan, and 35.5% in Indonesia [12].

There have been 5-year and 10-year health development projects in the health sector to ensure that Sustainable Development Goal (SDG) 2 -zero hunger- is met, while also working towards reducing poverty and inequality [14]. The inclusion of anemia reduction among WRA as an official target indicator for the second SDG2 in 2020 has created an opportunity for renewed dedication and focus on tackling this global public health issue. More than five years into the SDGs era, there is limited information on the global advancements toward the anemia target [15].

Even though there have been widespread attempts to halve the incidence of anemia by 2025, the prevalence remains alarmingly high, at 62.3% among reproductive-age women in Africa. In Mali, this condition affects 63% of women in this demographic group, with regions like Kayes experiencing severe cases at a rate of 78%, significantly higher than Kidal’s 48% [16–18]. In response, the Malian government has underscored the necessity for a multi-sectoral approach involving various governmental departments, including Agriculture, Health and Public Hygiene, Water and Sanitation, and Education [6].

To lessen the anemia burden in Mali, sufficient data regarding the role and contribution of variables at the individual, household, and community levels must be generated. However, only a few studies in the past explored anemia among women of reproductive age using the nationally representative Mali Demographic and Health Survey (MDHS) [6, 19] and malaria indicator survey [20]. As far as we are aware, none of these studies have conducted longitudinal analysis of anemia trends and its risk factors spanning nearly two decades among women of reproductive age in Mali, allowing for a more comprehensive understanding of how anemia prevalence has evolved. Therefore, this present study sought to assess the trends and factors associated with anemia among women of reproductive age in Mali using a multilevel analysis. Understanding these patterns and determinants may aid in developing targeted strategies and policies that will ensure Mali meets SDG 2 by 2030 and global targets by 2025. The study will also add to the practical dimension of public health implications and interventions as well as contribute to the extensive literature already written about anemia and provide the framework for future investigations into anemia-related problems in Mali.

## Materials and methods

### Data source and population study

Data from the 2001, 2006, 2012–2013, and 2018 MDHSs were used in this study. The MDHS, a national survey, is undertaken on a five-year basis to offer an important repository of data for in-depth analysis, examination of crucial aspects of fertility levels, maternal and child health, family planning, maternal and child nutrition, and newborn and child mortality in the country [21]. The MDHS was coordinated by the Planning and Statistics Unit of the Health, Social Development, and Family Promotion Sector, National Directorate of the Survey, and the National Institute of Statistics, Technical Directorate of the Survey [18]. The survey employs a two-stage stratified sampling technique, and the data captured is nationally representative. The first stage was characterized by the selection of clusters across urban and rural locations from the entire nation. These are made-up enumeration areas for the study. The second stage involved the selection of households from the predefined clusters from the previous population survey in Mali. The final reports contain information on the methodologies used in each of the survey rounds [18]. This is also available online at https://dhsprogram.com/data/dataset_admin/index.cfm. In this study, only women (*n* = 3,330 in 2001, 4,576 in 2006, 5,142 in 2012–2013, and 5,048 in 2018) were interviewed. After the addition of all data sets, the total number of respondents was 18,096. The study relied on ‘Strengthening the Reporting of Observational Studies in Epidemiology’ (STROBE) statements [22].

## Variables

### Dependent variable

This study investigated the presence of anemia among women of reproductive age in Mali using data from DHS’s anemia-level inquiry. Women of reproductive age in Mali who were willing underwent a hemoglobin test. The results were analyzed using a portable HemoCue analyzer.

This study used the WHO guidelines to define anemia as having a blood hemoglobin level [23]:

- i.e. none: if hemoglobin value is ≥ 12.0 g/dL.
- mild anemia: if hemoglobin value is from 11.0 to 11.9 g/dL.
- moderate anemia: if hemoglobin value is from 8.0 to 10.9 g/dL.
- severe anemia: if hemoglobin value is below 8.0 g/dL.

For this current study, the outcome variable was categorized as “not anemic = 0” (hemoglobin ≥ 12.0 g/dL) or “anemic = 1” (hemoglobin < 12.0 g/dL) [24].

### Independent variables

This study included eighteen independent variables, based on previous research [5, 6, 25, 26] and their availability in the datasets. These eighteen independent variables were further divided into three groups as variables of individual-level factors, household-level factors, and community-level factors.

### Individual level factors

Individual-level factors included age (15–19, 20–24, 25–29, 30–34, 35–39, 40–44, 45–49); educational level (no formal education, primary level, secondary/higher); marital status (never in union, married, cohabitation, widowed, divorced); employment status (not working, working); body mass index (BMI) (underweight, normal, overweight); pregnancy status (yes, no); and ever terminated pregnancy (yes, no). Another variable is parity. Parity was defined by the number of children a woman has ever given birth to. The responses were classified as zero, one, two, three, or four or more births.

### Household level factors

Household-level factors included mass media exposure, sex of the household head, wealth quintile, source of drinking water, type of toilet facility, and number of household members. Three factors were used to determine exposure to mass media. They were listening to the radio, watching television, and reading newspapers and magazines. These questions had the same response categories: “not at all,” “less than once a week,” and “at least once a week.” According to research [27], the answer options were divided into “no,” which indicated no mass media exposure (not at all), and “yes,” which signified mass media exposure (less than once a week and at least once a week). Household head’s sex (male, female) and number of household members (less than 5, 5 or more) were dummy variables; the wealth index was categorical: lowest, poorer, medium, richer, and richest.

The source of drinking water was characterized as enhanced (improved) or unimproved. Improved drinking-water sources are any water that, by its construction or active intervention, is likely to be protected against outside pollution, particularly fecal matter. Unimproved drinking water, on the other hand, is not protected against contamination; this includes unprotected wells, springs, rivers, dams, lakes, streams, ponds, rainwater, and others [28].

Toilet facilities were likewise divided into two categories: improved and unimproved. An improved toilet hygienically separates human excreta from direct contact with the body. Therefore, all flushes (pipe, septic, pit latrine), ventilated pit latrines, and composting toilets are in this category. Unimproved toilet types include pit latrines with slabs, pit latrines without slabs (open pits), no toilets, bushes, bucket toilets, hanging toilets, and others [29].

### Community-level variables

The community-level variables included four categories: region (Kayes, Koulikoro, Sikasso, Segou, Mopti, Toumbouctou, Gao, Kidal, Bamako); place of residence (urban, rural); community literacy level (low, medium, high); and community socioeconomic status (low, medium, high). The community literacy level was calculated based on the proportion of women who could read and write or could not read and write at all. Low community literacy included women in communities in the bottom 33rd percentile (less than 30% could read and write), while communities in the medium literacy level were those in the middle 33rd (between 30% and 70%), and those in the 33rd percentile (more than 70% could read and write) were in the high literacy level.

The community’s socioeconomic level was determined by the employment, income, and education of the women who lived there. We used principal component analysis to determine the number of women who were jobless, uneducated, and impoverished. A standardized score was created, with a mean of 0 and a standard deviation. The scores were then divided into three terciles: 1 (least disadvantaged), 2 (middle disadvantaged), and 3 (most disadvantaged), with tercile 1 reflecting a better socioeconomic situation and tercile 3 signifying a poorer one [5].

### Statistical analyses

The study used Stata version 14.2 to analyze the data from four recent rounds of the MDHS and investigate the association between anemia and characteristics of women of reproductive age. Descriptive analysis and multivariate regression models were used to investigate the distribution of anemia status and independent factors. Pearson’s (chi-square) test was utilized to analyze correlations between anemia and explanatory factors. Multilevel model findings were stated as adjusted odds ratios and 95% confidence intervals. A forest plot was used to show the risk and protective factors of anemia. We checked for high correlation among the independent variables using the variance inflation factor for variables (VIF test). The results showed no evidence of high collinearity (mean VIF = 1.51, maximum = 2.50, minimum = 1.02). The maximum VIF is place of residence, and the minimum VIF is pregnancy status.

To study the risk and protective factors of anemia, a multilevel regression was performed on a pooled dataset. The first model, designated as Model 0, was the null model; it had only the dependent variable (anemia), but because of the main sample units’ distribution, it revealed variation in the outcome variable. Only individual-level components were present in Model 1, whereas only household-level factors were included in Model 2. Only community-level characteristics were considered in Model 3. The complete model, which is model 4, was a comprehensive model with the full range of variables accounted for.

In this study, employing Akaike’s Information Criterion (AIC) for model selection is fitting, especially in the framework of a multilevel analysis utilizing DHS data. The AIC emphasizes predictive accuracy by penalizing excessively complex models, thereby mitigating the risk of overfitting while still capturing significant relationships in the data. While other metrics, such as the Bayesian Information Criterion (BIC), focus more on identifying the “true” model and are better suited for smaller samples, AIC’s emphasis on predictive performance aligns with the aims of this research. Moreover, AIC facilitates the comparison of both nested and non-nested models, granting flexibility in model selection. By utilizing AIC, this study adopts a rigorous and transparent model selection process, improving the reliability and clarity of the findings. As a result, the study’s lowest AIC was used for the discussion. The research sample was weighted using v005/1,000,000 and analyzed using Stata’s survey set command (svy) to account for the survey’s complexity and the results’ generalizability. We restricted our analysis to complete cases; therefore, all missing values were dropped.

## Results

### Trends of anemia from 2001 to 2018

**Fig. 1:**
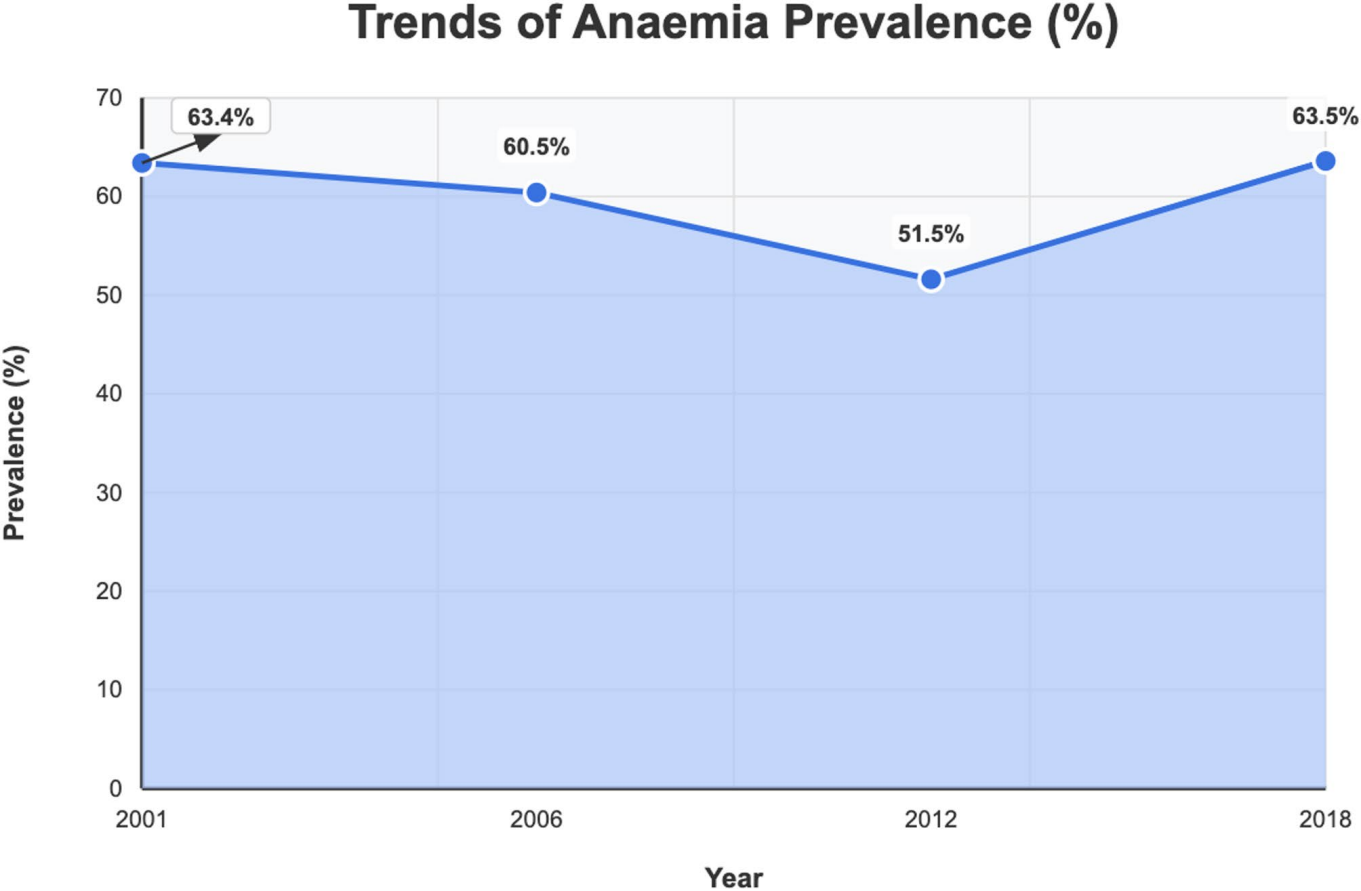
Trends of anemia from 2001 to 2018 in Mali Source: MDHS 2001, 2006, 2012, and 2018

According to the data, the anemia trend among women of reproductive age in Mali indicated a decline from 63.4% in 2001 to 60.4% in 2006. The proportion of those suffering from anemia declined to 51.5% in 2012 and then grew to 63.5% in 2018. (see Fig. 1).

### Prevalence of anemia from 2001 to 2018

**Fig. 2:**
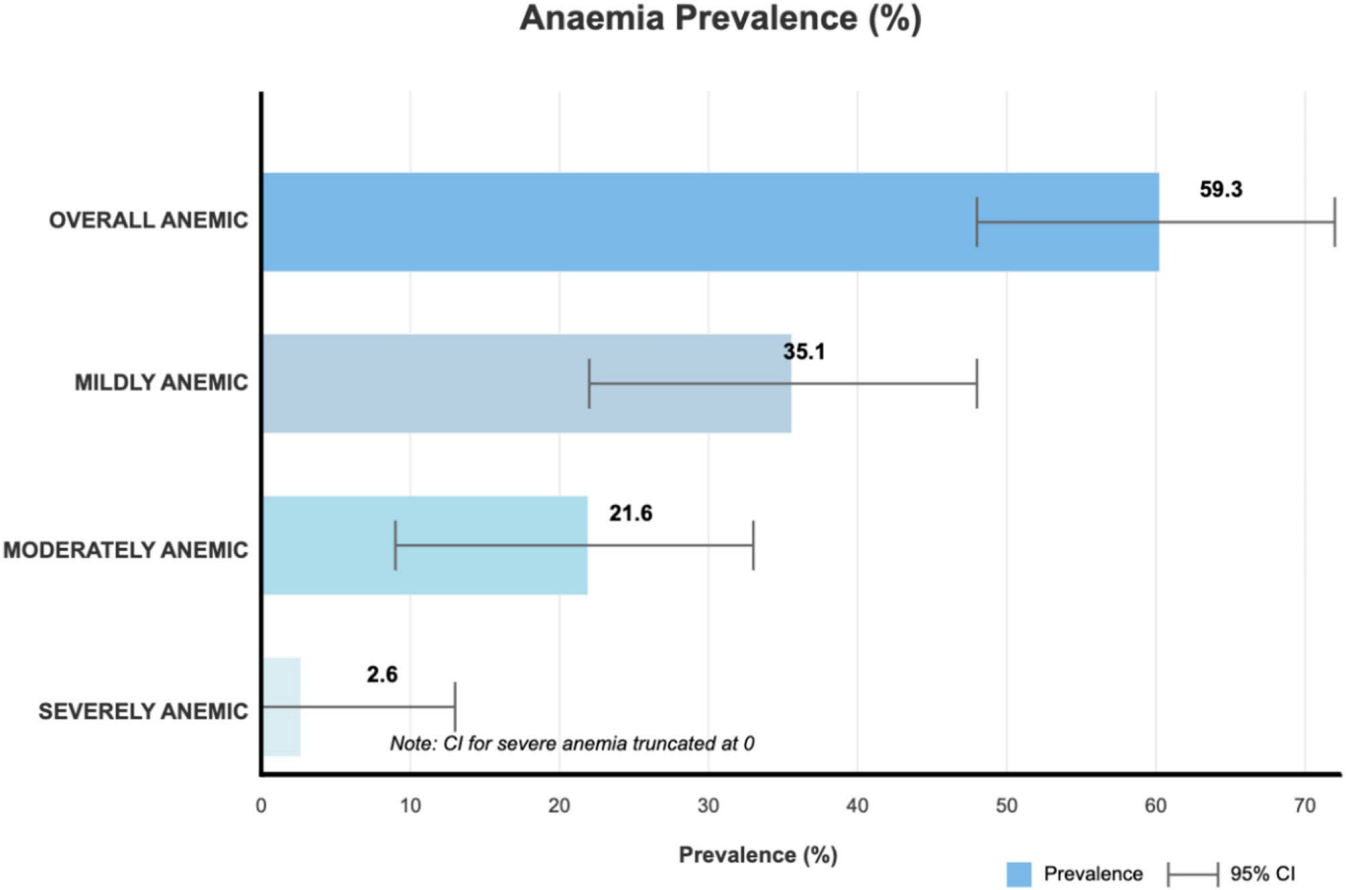
Average prevalence of anemia from 2001 to 2018 among women of reproductive age (%) Source: MDHS 2001, 2006, 2012, and 2018

From 2001 to 2018, the overall pooled data showed that 59.3% [95% CI: 58.2-60.4%] of women of reproductive age in Mali were anemic. Of them, 21.6% [95% CI: 20.6-22.6%] and 35.1% [95% CI: 34.1-36.1%] were anemic in the moderate to mild range, while 2.6% [95% CI: 2.3-2.9%] were seriously anemic. (see Fig. 2).

### Distribution of anemia across the independent variables from 2001 to 2018

Table 1 depicts the results of the distribution of anemia among women of reproductive age in Mali between 2001 and 2018 across the independent variables. The findings suggest that the variables of educational status, marital status, pregnancy status, BMI, mass media exposure, region, wealth quintile, source of drinking water, type of toilet facility, community literacy level, community socioeconomic status, and survey years had *p*-values of 0.000. Whereas the variables of parity and employment had 0.010 and 0.037, respectively.

Nonetheless, the *p*-values for the maternal age, terminated pregnancy, sex of household head, and number of household members were 0.115, 0.730, 0.320, and 0.959, respectively.

**Table 1:** Distribution of anemia across the independent variables from 2001–2018 (*N* = 18,096)

Variables	Anemia Prevalence (%)		X^2^	*p*-value
	No (%)	Yes (%)		
**Individual-level**				
**Women’s age (years)**			**10.23**	**0.115**
15–19	40.8	59.2		
20–24	41.4	58.6		
25–29	41.6	58.4		
30–34	41.3	58.7		
35–39	40.0	60.0		
40–44	38.3	61.7		
45–49	39.2	60.8		
**Women’s educational level**			**193.77**	0.000*
No education	37.9	62.1		
Primary	44.7	55.3		
Secondary+	52.8	42.3		
**Marital status**			**29.18**	0.000*
Never in union	45.6	54.4		
Married	39.8	60.2		
Cohabiting	42.9	57.1		
Widowed	46.9	53.1		
Divorced	40.2	59.8		
**Women’s occupational status**			**4.35**	0.037*
Not working	41.0	58.9		
Working	40.4	59.6		
**BMI**			**201.25**	0.000*
Underweight	36.5	63.5		
Normal	38.7	61.3		
Overweight	50.1	49.9		
**Parity**			**37.22**	0.010*
0 birth	43.5	56.5		
1 birth	42.0	58.0		
2 births	43.3	56.7		
3 births	40.2	59.8		
> 3 births	38.6	61.4		
**Pregnancy status**			**89.41**	0.000*
No	42.0	58.0		
Yes	31.9	68.1		
**Terminated Pregnancy**			**16.33**	**0.730**
Yes	41.0	59.0		
No	38.8	61.2		
**Household-Level**				
**Mass media exposure**			**38.70**	0.000*
No	37.8	62.3		
Yes	42.3	57.7		
**Sex of household head**			**0.99**	**0.320**
Male	40.6	59.4		
Female	41.4	58.6		
**Wealth quintile**			**320.11**	0.000*
Poorest	34.5	65.5		
Poorer	37.0	63.0		
Middle	37.7	62.4		
Richer	41.7	58.3		
Richest	51.6	48.4		
**Source of drinking water**			**136.26**	0.000*
Unimproved	35.2	64.8		
Improved	44.0	56.0		
**Type of toilet facility**			**52.02**	0.000*
Unimproved	35.3	64.7		
Improved	41.6	58.4		
**Number of household members**			**0.00**	**0.959**
Less than 5	40.8	59.2		
5+	40.7	59.3		
**Community-level**				
**Region**			**273.41**	0.000*
Kayes	34.9	65.1		
Koulikoro	40.1	59.9		
Sikasso	40.8	59.2		
Segou	42.3	57.7		
Mopti	34.9	65.1		
Toumbouctou	37.4	62.6		
Gao	36.8	63.2		
Kidal	80.9	19.1		
Bamako	51.6	48.4		
**Place of residence**			**279.38**	0.000*
Urban	49.4	50.6		
Rural	37.5	62.5		
**Community literacy level**			**342.92**	0.000*
Low	32.9	67.1		
Medium	41.4	58.6		
High	49.4	50.6		
**Community socioeconomic status**			**268.44**	0.000*
Low	36.4	63.6		
Moderate	40.4	59.6		
High	48.6	51.4		
**Survey year**			**174.82**	0.000*
2001	36.7	63.4		
2006	39.5	60.5		
2012	48.5	51.5		
2018	36.5	63.5		

A *p*-value below 0.05 shows statistical significance

Source: MDHS 2001, 2006, 2012 and 2018

The highest proportion of anemia (61.7%) was found in women aged 40–44, whereas the lowest prevalence (58.4%) was seen in the 25–29 years’ group. Women who had secondary and higher formal education had a prevalence of anemia equal to 42.3%; those women of reproductive age with no education had the highest proportion (62.1%). Furthermore, compared to women of reproductive age who were never in union (54.4%) and did not work (58.9%), married women (60.2%) and working women (59.6%) had the greatest proportion of anemia. Women of reproductive age who were overweight (49.9%), women with no birth (56.5%), and women who had mass media exposure (57.7%) had lower anemia prevalence.

The largest percentage of anemic women were identified in the poorest wealth quintile (65.5%), the Mopti region (65.1%), male-headed households (59.4%), and the majority of never-terminated pregnancies (61.2%). The highest anemia prevalence was found in women who had unimproved sources of drinking water (64.8%), unimproved types of toilet facilities (64.7%), five or more household members (59.3%), lived in rural areas (62.5%), were in communities with low literacy levels (67.1%), were in communities of low socioeconomic status (63.6%), and were included in the 2018 survey (63.5%).

### Forest plot of key risk and protective factors for anemia among women of reproductive age in Mali from 2001 to 2018

**Fig. 3:**
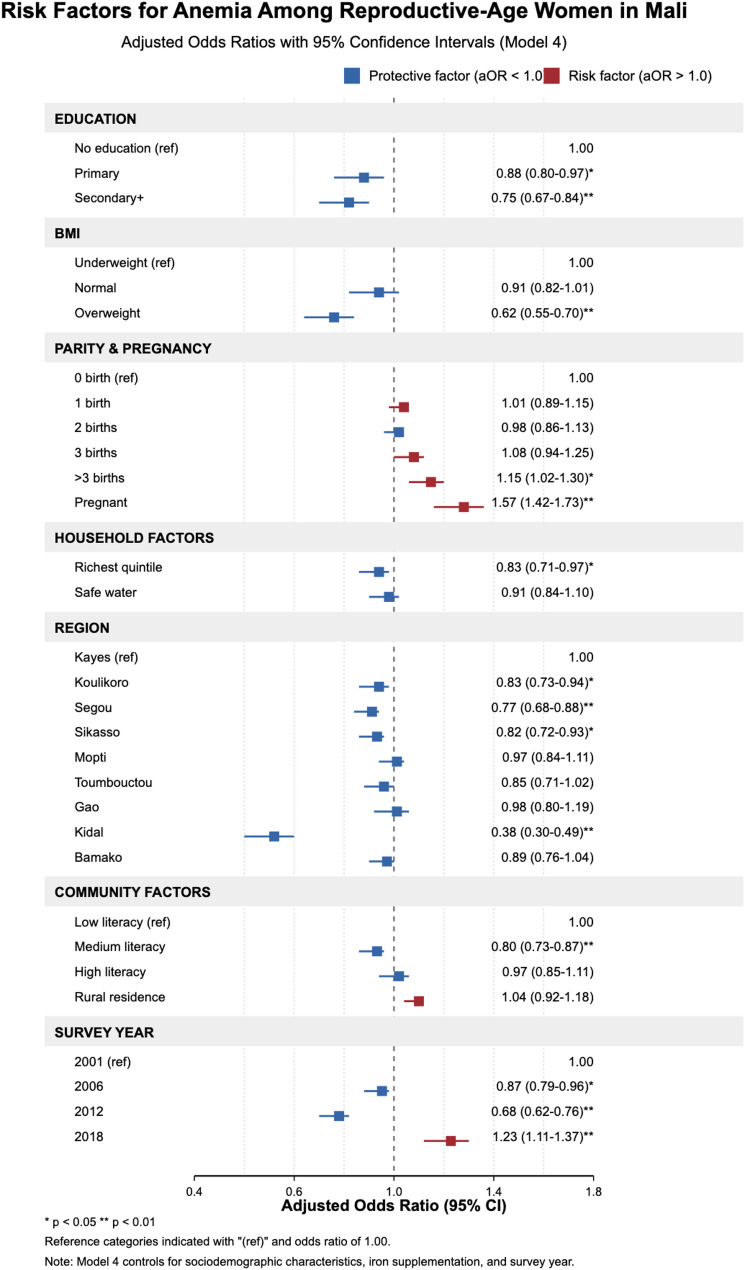
Forest plot of key risk and protective factors for anemia among women of reproductive age in Mali

Figure 3 presents the results of the forest plot of key risk and protective factors for anemia among women of reproductive age in Mali from 2001 to 2018. The results show a decreased risk of anemia among women of reproductive age who had formal education. Those with secondary education and beyond had lower risks of becoming anemic [aOR = 0.75; 95% CI = 0.67–0.84] compared to those without formal education. Comparing overweight and underweight among women of reproductive age, anemia was less likely in the former group [aOR = 0.62, 95% CI = 0.55–0.70]. Women of reproductive age with three or more children had higher risks of anemia. Specifically, women with four or more births had an aOR of 1.15 (95% CI: 1.02–1.30) higher than those who had zero births. The risk of anemia was higher in pregnant women than in non-pregnant ones (aOR = 1.57, 95% CI = 1.42–1.73). The risk of being anemic is reduced with rising wealth status. Women whose households were categorized in the richest wealth quintile were almost 20% less likely to develop anemia [aOR = 0.83, 95% CI = 0.71–0.97] than women whose households belonged to the poorest wealth quintile. Women with access to improved sources of drinking water [0.91, 95% CI = 0.84–1.10] were less likely to be described as anemic.

Compared to women of reproductive age who lived in the Kayes Region, women who lived in the Kidal Region were less likely to be classified as having anemia [aOR = 0.38, (95% CI = 0.30–0.49)]. With regard to community literacy, compared to women in communities with low literacy levels, those in communities with medium literacy levels had a lower risk of anemia [aOR = 0.80, 95% CI = 0.73–0.87]. Compared to women interviewed in the 2001 survey year, those included in the 2018 survey year had a 23% higher likelihood of being anemic [aOR = 1.23, 95% CI = 1.11–1.37].

Other variables, such as occupational and marital status, mass media exposure, type of toilet facility, place of residence, and community socioeconomic status, did not show any statistically significant correlation with anemia.

### Random effects (variance measures) results

In comparison to the final model (Model Four), Table 2 demonstrates that AIC values reduced in models One, Two, and Three with the key independent variables, individual-household-community-level variables. The significant drop of AIC values confirms that Model Four, which contained all the independent variables, had the best fit. The complete Model 4 was used to predict anemia risk variables among women of reproductive age in Mali.

The parameters linked to anemia among women of reproductive age in Mali changed significantly during the PSU clustering in the Zero model (σ2 = 0.14, 95% CI 0.11–0.18). The between-cluster variability in the variables explained 0.041 of the variances in anemia drivers among women of reproductive age, based on the ICC finding for Model Zero. The individual-level component of Model One reduced the difference between clusters to 0.031. The ICC decreased to 0.027 in Model Two, which only included covariates at the household level. The ICC then rose to 0.021 in Model Three, which only included community-level factors. Model Four, the last model, reduced the variation between clusters to 0.020. Changes in the way the data are clustered can help to explain this.

**Table 2:** Random effects (variance measures) results anemia among women of reproductive age in Mali from 2001 to 2018

Random effect results	Model Zero	Model One aOR (95% CI)	Model Two aOR (95% CI)	Model Three aOR (95% CI)	Model Four aOR (95% CI)
PSU variance (95% CI)	0.14 (0.11–0.18)	0.10 (0.08–0.14)	0.09 (0.07–0.13)	0.07 (0.05–0.10)	0.07 (0.05–0.10)
ICC	0.041	0.031	0.027	0.021	0.020
Wald chi-square	Ref	390.24	269.68	557.09	769.76
LR test	X^2^ = 174.98, *p* < 0.001	X^2^ = 102.77 *p* < 0.001	X^2^ = 84.81, *p* < 0.001	X^2^ = 53.43, *p* < 0.001	X^2^ = 51.31, *p* < 0.001
**Model fitness**					
Log-likelihood	-12170.2	-11971.3	-12038.3	-11893.5	-11774.2
AIC	24344.4	23974.5	24094.7	23823.0	23626.4
BIC	24360.0	24099.4	24164.9	23963.5	23930.7
**N**	18,096	18,096	18,096	18,096	18,096
**Number of groups**	533	533	533	533	533

Source: 2001–2018 MDHSs. AIC- Akaike’s Information Criterion; aOR - adjusted odds ratio; BIC- Bayesian Information Criterion; ICC - Intra-Class Correlation Coefficient; PSU - Primary Sampling Unit

## Discussion

This study aimed at assessing the trends and factors associated with anemia among women of reproductive age in Mali. In this study, it was found that anemia has marginally increased over the years. It was seen from the findings of this study that anemia decreased from 63.4% in 2001 to about 51.5% in 2012. However, after that, it increased to the highest level since the beginning of the 21st century, to about 63.5% in 2018. This finding is consistent with an earlier study conducted in Uganda [30]. According to Nankinga and Aguta [30], anemia among women of reproductive age declined significantly from 50% in 2006 to 23% in 2011. Nonetheless, the prevalence then increased to 32% in 2016. A possible reason could be associated with the high prevalence of malaria and high fertility rate, coupled with limited healthcare facilities. Another possible reason may be related to the presence of vaginal and parasitic infections, along with culturally imposed dietary restrictions [31, 32]. Therefore, addressing and managing these infections, together with providing health education about proper nutrition during pregnancy, could help reduce anemia in Mali and other nearby countries facing similar health challenges [12].

We found that from 2001 to 2018, the overall anemia prevalence among women of reproductive age in Mali was 59.3% [95% CI: 58.2–60.4%]. From the pooled analysis, about 6 out of 10 women of reproductive age experienced anemia between 2001 and 2018. Despite the various prevention strategies that have been instituted by the Malian government through various developmental bodies since 2001, anemia prevalence continues to be a serious public health concern. The anemia rate presented in this study is greater than 34.85% in Eastern Africa [11], 38% in Congo DR [33], 41% in Nepal [34], 52.6% in South and Southeast Asia [35], 41.7% in Bangladesh [36], and 41.74% in sub-Saharan Africa [37]. However, compared to studies in Mali (63.5%) [6] and Pakistan (77%) [35], anemia prevalence among women of reproductive age was found to be lower in this current study. Anemia prevalence discrepancies found might be caused by geographical locations, nutritional, and cultural variables [11]. Furthermore, Mali’s high prevalence of anemia among women of reproductive age may be due to their intrinsic and cultural sensitivity to anemia. Furthermore, due to poor socioeconomic position, underutilization, and insufficient health care access, women of reproductive age in Mali may have limited access to treated insecticide mosquito nets and iron-rich diets [6].

In this study, among women of reproductive age in Mali, 2.6% had severe anemia, compared to moderate anemia of 21.6% and mild anemia of 35.1%. The severity of anemia among women of reproductive age in Mali in this study is higher than 2.3%, 2%, 1.6%, and 1.4% reported in other studies in Mali [20], Pakistan [38], Tanzania [39], and Nigeria [40], respectively. Conversely, the severity of anemia among women of reproductive age in Mali in this research is less than 4.3% and 12.2% reported in Mali [6] and Senegal [41].

The study’s multivariate regression analysis revealed that a variety of variables are associated with anemia among women of reproductive age in Mali. Anemia was found to be significantly correlated with maternal education level, parity, pregnancy status, wealth quintile, BMI, source of drinking water, region, community literacy level, and survey year. The results of this investigation are in line with earlier studies from Eastern Africa [11], Nigeria [20], Uganda [30], and India [42, 43].

It was determined through this analysis that maternal education is a crucial determinant of anemia among women of reproductive age. Reproductive-age women with at least primary education have a decreased risk of anemia, presumably due to their understanding of good hygiene practices, healthcare decision-making autonomy, and adopting healthy lifestyles that may include consuming nutritious food [6, 20]. This conclusion is supported by the results of studies conducted in LMICs [12, 42, 44–47]. This emphasizes the significance of educational interventions in combating anemia among women of reproductive age in Mali, since formal education enables women of reproductive age to make informed health and nutrition decisions. Hence, it is essential for public administrators in Mali to implement a policy that ensures universal access to formal education [48].

This study at hand suggests that being overweight can lower the risk of developing anemia among women of reproductive age in Mali. This outcome is in line with several other studies from Mali [6], Nepal [34], and Bangladesh [48]. This might be because overweight women may have consumed more iron and had appropriate and healthier diets [49–51]. Another possible reason is that underweight women may have had other comorbidities, including acute respiratory illness, malaria, diarrhea, and bacteremia [49, 51]. This calls for policy intervention that will prioritize essential micronutrients among women of reproductive age in Mali.

In Mali, we discovered that women of reproductive age who had at least one birth are at higher risk of being anemic [6, 49]. According to the analysis of this current study, women who have three or more births are at greater odds of being anemic. Similar results have been obtained in LMICs [44], Ethiopia [47, 52, 53], India [54], Indonesia [55], and Nepal [56]. Due to exposure to blood loss, having more children might result in greater iron loss, underscoring the importance of the utilization of contraceptives [57].

During pregnancy, there is blood expansion, and this tends to increase iron and folic acid demand among women [33]. Therefore, when the woman’s body can produce the smallest amount of red blood cells to avoid anemia, a large amount of blood is needed to support the growth of the unborn baby. Therefore, it’s crucial to remember that a woman’s pregnancy is the period of her life when she needs to consume the most nutrients. Hence, it is recommended for pregnant women to have a more diverse diet than usual [57]. During pregnancy, the WHO has recommended that as early as possible, women must start taking 0.4 mg of folic acid and 30–60 mg of iron supplements [35]. This study suggests that anemia is more common among pregnant women. Comorbidities associated with pregnancy, such as diabetes, hypertension, and intestinal infections, may be the cause of high anemia in expectant mothers [39]. The finding of this current study is in tangent with earlier studies done in Uganda [30], Senegal [41], and Nepal [34].

The results from the study also revealed that a wealth quintile was seen as a determinant of anemia in women of reproductive age. The study found that women in the richest wealth quintile were less likely to be classified as having anemia, a condition linked to poor socioeconomic status, indicating a potential risk factor for malnutrition [27, 33]. The results of similar research carried out throughout Eastern Africa [12, 58, 59], West Africa [6, 20], and Asia [49, 60] support the finding from this study. The possible reason is that women in the richest wealth quintile possess the financial capacity to purchase high-quality foods. On the other hand, a study in Nepal revealed that there was no statistically significant correlation between wealth index and anemia among women of reproductive age [34].

As observed in previous studies in Mali [6] and Uganda [33], a source of drinking water was a significant determinant of being anemic among women of reproductive age. The possible reason could be that women of reproductive age using unimproved sources of drinking water may have been more susceptible to anemia due to exposure to waterborne illnesses through the open source of the water [11, 33].

The region of residence of women of reproductive age in Mali was found to be a significant determinant. Findings from studies conducted in Mali [6], Nepal [60], and Ecuador [61] showed that region and community literacy levels are significantly associated with anemia. This current study found that women who stayed in the Kidal region were less likely to be categorized as having anemia than those who lived in the Kayes region. A few studies have shown that dietary habits, the accessibility, availability, and distance to medical facilities, and iron supplements may affect the risk of anemia in different regions [6, 62, 63]. Also, the Kidal region, which is situated closer to the Sahara Desert, experiences a much drier climate than the Kayes region, which is in the more humid western part of Mali. This difference in climate likely leads to a reduction in malaria transmission, which significantly contributes to anemia in the Kidal region [64, 65]. Additionally, high fertility rates and frequent pregnancies can lead to a depletion of iron reserves, heightening the risk of anemia. Women in the Kidal region may have lower fertility rates than those in the Kayes region due to cultural or socio-economic factors. Mali’s DHS reports indicate notable regional variation in fertility rates, with northern areas such as Kidal typically having lower rates compared to the southern and western regions such as Kayes [18].

Finally, community literacy level was another statistically significant variable that is connected to anemia prevalence among women of reproductive age. In comparison to their counterparts in low-literacy-level communities, women in medium- and high-literacy-level communities also displayed a lower proportion of anemia. Communities with medium or high literacy levels are argued to include women of reproductive age who have received formal education and are employed. Women in this setting are financially empowered to afford healthcare and safe drinking water. The results are in line with a similar study conducted in West Africa [6].

### Strengths and limitations

The main strength of this study is that it is the first to assess the trends and factors associated with anemia in reproductive age using nationally representative data from 2001 to 2018 in Mali. This study also allows for the generalization of the findings to other women in Mali using a rigorous, scientifically sound methodological approach. Despite these strengths, the study has some weaknesses that need to be recognized. First, the study relied on secondary data, and data for the analysis were limited to the variables found in the Malian dataset. Hence, the interpretation should be limited to the variables that were used in this study. Also, the cross-sectional nature of the study does not allow for causality to be inferred from the findings. Additionally, several factors, including dietary intake and parasitic and genetic hemoglobin conditions, were not presented in the datasets. In Mali, malaria is a major cause of anemia, especially in areas with high transmission rates, and not including it may lead to an underestimation of its impact on anemia rates, obscure regional differences, and create confounding variables. Hence, future studies should aim to incorporate these variables and perform sensitivity analyses to gain a deeper insight into the causes of anemia and to aid in formulating effective strategies to decrease anemia prevalence among women of reproductive age in Mali.

### Conclusion and implications for public health policy

Our study revealed a slight increase in anemia from 2001 to 2018. There is also a high prevalence of anemia among women of reproductive age. Women’s educational level, BMI, pregnancy status, wealth index, parity, source of drinking water, region, and community literacy are associated with the prevalence of anemia. It is imperative, therefore, that the policymakers consider these factors when designing health and nutrition interventions and programs that would mitigate anemia prevalence among women of reproductive age in Mali. These interventions and programs should include strengthening antenatal care (ANC) services for pregnant women through the provision of free maternal health care, the provision of iron supplements, treated mosquito nets, and deworming.

Additionally, the government of Mali and its agencies, such as the Ministry of Education, should prioritize women’s education, particularly secondary education completion, which is essential as it equips women with the knowledge to prevent anemia. This will empower women and help find solutions to poverty and inequality. Furthermore, the Ministry of Water and Sanitation, traditional leaders, and authorities should take appropriate measures to ensure that every household has access to an improved source of drinking water through infrastructural development.

Rural regions in Mali frequently face challenges such as inadequate healthcare facilities, insufficient trained staff, and limited access to clean water. Given Mali’s dependence on international assistance, the country should engage international organizations and donors in supporting comprehensive anemia reduction initiatives. This can impact socio-economic aspects, including wealth indices and community literacy levels, through tackling poverty alleviation programs and community education initiatives.

Targeted interventions reflecting regional variations in anemia prevalence should be developed, ensuring effective resource allocation. Cross-sectoral collaboration across various sectors, including the Ministry of Health, Ministry of Education, Ministry of Water and Sanitation, traditional leaders, and other stakeholders, is vital for the comprehensive implementation of these strategies. By addressing the interconnected elements—such as education, healthcare access, clean water, and socioeconomic inequalities—policymakers can create effective measures that not only lower anemia rates but also enhance overall health outcomes for women of reproductive age in Mali.

## Data Availability

No datasets were generated or analysed during the current study.

## Abbreviations

AIC: Akaike’s Information Criterion (AIC)
ANC: Antenatal Care
aOR: Adjusted Odds Ratio
BMI: Body mass index
CI: Confidence interval
DHS: Demographic and Health Surveys
EAs: Enumeration Areas
ICC: Intra-Class Correlation Coefficient
LMICs: Low- and Middle-Income Countries
MDHS: Mali Demographic and Health Survey
PSU: Primary Sampling Unit
SDGs: Sustainable Development Goals
VIF: Variance Inflation Factor
WHO: World Health Organization
WRA: Women of Reproductive age
